# Ocular blood flow evaluation by laser speckle flowgraphy in pediatric patients with anisometropia

**DOI:** 10.3389/fpubh.2023.1093686

**Published:** 2023-02-27

**Authors:** Takashi Itokawa, Tadashi Matsumoto, Saiko Matsumura, Momoko Kawakami, Yuichi Hori

**Affiliations:** Department of Ophthalmology, School of Medicine, Toho University, Tokyo, Japan

**Keywords:** laser speckle flowgraphy, amblyopia, anisometropia, ocular blood flow, reproducibility, optic nerve disk, choroid

## Abstract

**Purpose:**

To determine the differences and reproducibility of blood flow among hyperopic anisometropic, fellow, and control eyes.

**Methods:**

We retrospectively studied 38 eyes of 19 patients with hyperopic anisometropia (8.2 ± 3.0 years of age) and 13 eyes of eight control patients (6.8 ± 1.9 years). We measured the optic nerve head (ONH) and choroidal circulation using laser speckle flowgraphy (LSFG) and analyzed the choroidal mean blur rate (MBR-choroid), MBR-A (mean of all values in ONH), MBR-V (vessel mean), MBR-T (tissue mean), and sample size (sample), which are thought to reflect the ONH area ratio, area ratio of the blood stream (ARBS). We then assessed the coefficient of variation (COV) and intraclass correlation coefficient (ICC) and compared the differences among amblyopic, fellow, and control eyes in MBR, sample, and ARBS.

**Results:**

The ONH, MBR-A, MBR-T, and ARBS of amblyopic eyes were significantly higher than those of fellow eyes (*P* < 0.01, *P* < 0.05, and *P* < 0.05, respectively), and control eyes (MBR-A and ARBS, *P* < 0.05, for both comparisons). The sample-T (size of tissue component) in amblyopic eyes was significantly smaller than that in fellow and control eyes (*P* < 0.05). Blood flow in the choroid did not differ significantly between the eyes. The COVs of the MBR, sample, and ARBS were all ≤10%. All ICCs were ≥0.7. The COVs of pulse waveform parameter fluctuation, blowout score (BOS), blowout time (BOT), and resistivity index (RI) in the ONH and choroid were ≤10%.

**Conclusion:**

The MBR value of the LSFG in children exhibited reproducibility. Thus, this method can be used in clinical studies. The MBR values of the ONH in amblyopic eyes were significantly high. It has been suggested that measuring ONH blood flow using LSFG could detect the anisometropic amblyopic eyes.

## 1. Introduction

The increasing number of patients with refractive error, a known risk factor for amblyopia, has attracted worldwide attention (1). If amblyopia due to refractive error is not treated at the appropriate time in childhood, good vision will not be achieved, resulting in amblyopia. The prevalence of amblyopia is related to income level, age, ethnicity, public awareness, and screening programs; specifically, amblyopia has shown higher prevalence in people with low income, aged over 20 or under 10 years, and located in Europe, Oceania and North America (2). A recent study reported that amblyopia prevalence will increase from 99 million in 2019 to 221 million in 2040 (2). Myopia is more common in Asia, while amblyopia is more common in Europe and North America. Although the distribution of refractive error varies across regions, management of childhood refractive error is becoming increasingly important (3). The prevalence of amblyopia is reported to be 0.74–4.3%, and the most frequent form is anisometropic amblyopia (3, 4).

Although anisometropic amblyopia occurs when differences in refractive values between eyes cause developmental disorders, resulting in one eye being amblyopic, it has been reported that there are also differences in ocular structure between the right and left eyes (5–7). In patients with anisometropic amblyopia, the amblyopic eye exhibits a shorter axial length, smaller optic nerve head (ONH) diameter, and thicker choroid (5, 6). In previous studies pulsatile ocular blood flow (POBF) and color Doppler ultrasonography have been used to evaluate retrobulbar blood flow in anisometropic amblyopic eyes and reported that blood flow between amblyopic and fellow eyes did not differ significantly (8, 9).

Laser speckle flowgraphy (LSFG) is a non-invasive technique for measuring ocular blood flow (10–13), and the mean blur rate (MBR) is an indicator of ocular blood flow (14). Many investigators have used LSFG to measure ocular blood flow in patients with glaucoma (14, 15), retinal vascular occlusion (16), or diabetic retinopathy (17). LSFG has also been used to study the relationship between ocular blood flow and systemic diseases such as sleep apnea syndrome and chronic kidney disease (18, 19). A recent study also reported that MBR and age were significantly correlated, and females have higher MBRs than males (20). However, to the best of our knowledge, there are no published studies on blood flow using LSFG in patients with anisometropic amblyopia other than case reports (21). We hypothesized that differences in ocular structure in patients with anisometropia also affect ocular hemodynamics. The purpose of the present study was to investigate the differences in ocular blood flow attributable to differences in ocular structure among amblyopic, contralateral, and control eyes after assessing the reproducibility of the LSFG measurement value.

## 2. Methods

### 2.1. Patients

This was a retrospective, cross-sectional observational study, and all patients visited Toho University Omori Medical Center between April 2015 and July 2022. This study was approved by the Ethics Committee of Toho University Omori Medical Center (#M22161) and registered in the University Hospital Medical Information Network (UMIN) (Registry No. UMIN000049300). This study adhered to the tenets of the Declaration of Helsinki. This study was presented on our institutional website and the right to opt out was provided to all parents. This retrospective study comprised 19 amblyopic eyes and their fellow eyes of 19 pediatric patients with hyperopic anisometropic amblyopia [12 males and seven females; 5–15 years of age; 8.2 ± 3.0 years (mean ± standard deviation (SD))] and 13 eyes of 8 pediatric control patients (five males and three females; 5–10 years of age; 6.8 ± 1.9). Hyperopic anisometropic amblyopia was defined as an interocular difference in the cycloplegic spherical equivalent (SE) of 2.00 diopters (D) between the amblyopic and fellow eyes. Moreover, patients with anisometropic amblyopic had a best-corrected visual acuity (BCVA) of 20/20 or better vision due to treatment and did not have strabismus. Pediatric control patients who matched the axial length to the amblyopic eye were defined as those with a visual acuity of 20/20 or better vision and did not have strabismus, anisometropic amblyopia, history of intraocular surgery, cataract, glaucoma, or retinal disorder. We excluded patients who were not cooperative enough for the LSFG examination.

### 2.2. LSFG examination

**Figure 1:**
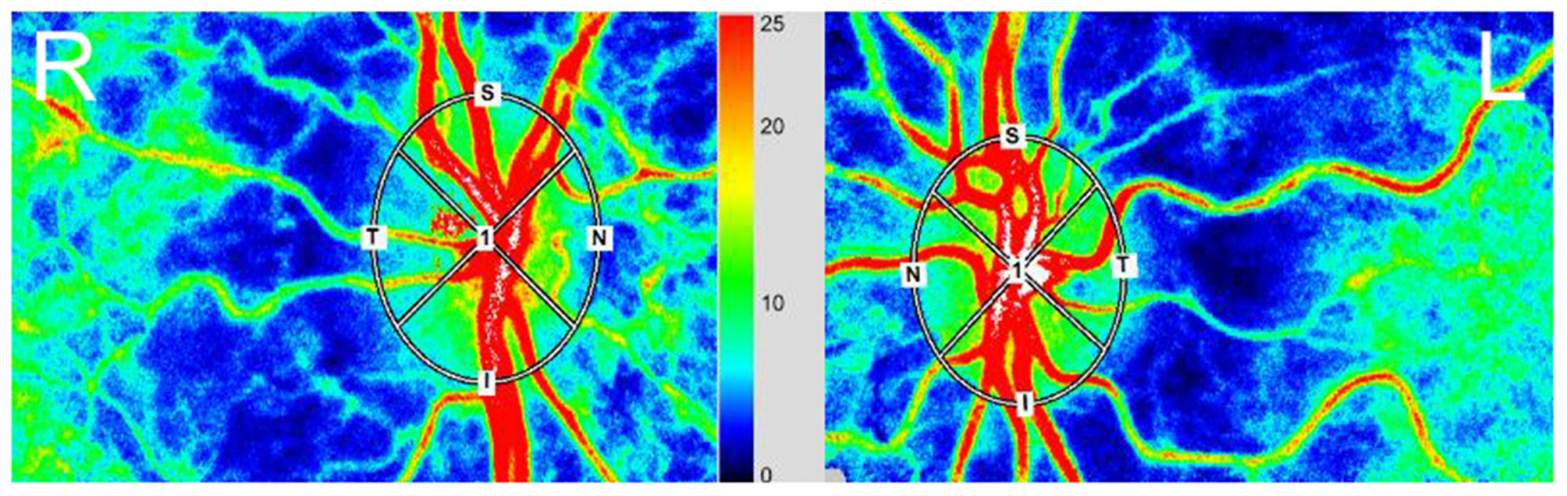
Red, high blood flow; Blue, low blood flow. The margin in the optical nerve head (ONH) was determined manually using a rubber band. **(Right)** eye (R), fellow eye. **(Left)** eye (L), amblyopic eye.

**Figure 2:**
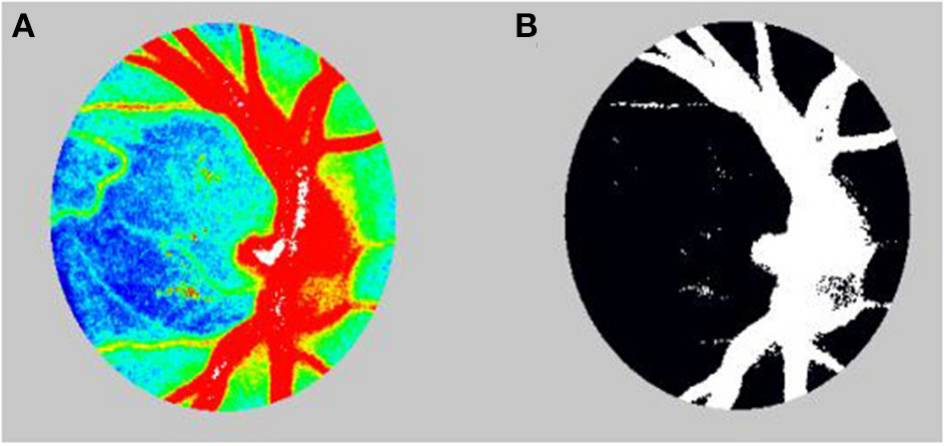
**(A)** Color-coded map before calculating the area ratio of the blood stream (ARBS). **(B)** In calculation of the ARBS, the vessels were separated using the automated definitive threshold.

Although we used the LSFG-baby, a modified version of LSFG that enables measurements with the subject in a supine position, to measure blood flow at the ocular fundus in neonates (22, 23), LSFG was performed using the LSFG-NAVI™ (Nidek, Aichi, Japan) in this study. Before examination, the patient's pupils were dilated with 0.4% tropicamide. The LSFG measurement method has been previously described in detail (10, 24). The measurements were conducted three consecutive times, and the ONH and choroid areas were analyzed. All measurements were performed by the same examiner (TI). The LSFG used the MBR as an indicator of blood flow. After the margin of the ONH was manually set (Figure 1), we calculated the MBR and number of samples in the ONH using LSFG Analyzer software (v3.8.0.4; Softcare, Fukuoka, Japan). For the choroidal blood flow (MBR-choroid), a rectangular area (200 × 200 pixels) was analyzed between the fovea and ONH, avoiding large retinal vessels. We also divided the MBR-all (MBR-A: the mean of all values) in the ONH into the MBR-vessel (MBR-V: component of vessels in the ONH) and MBR-tissue (MBR-T: component of tissues in the ONH) and calculated these three parameters. We assumed that the number of measurement points reflects the ONH area and defined the number of measurement points in the ONH as the sample size, which is equal to the pixel size. We divided sample-all (Sample-A: the mean of all sample sizes) in the ONH into sample-vessel (Sample-V: size of vessel component) and sample-tissue (Sample-T: size of tissue component) and calculated these three parameters. The area ratio of the bloodstream (ARBS, %) was defined as the ratio of the vessel area in the ONH. The vessel area was separated using an automated definitive threshold (Figure 2) (17). Nine pulse waveform parameters were calculated: fluctuation, skew, blowout score (BOS), blowout time (BOT), rising rate, falling rate, flow acceleration index (FAI), acceleration time index (ATI), and resistivity index (RI) (24).

### 2.3. Analysis of reproducibility

To determine intra-examiner reproducibility when measured three consecutive times, we assessed the reproducibility of the MBR, number of samples, ARBS, and nine pulse waveform parameters by determining the coefficient of variation (COV) and intraclass correlation coefficient (ICC).

### 2.4. Other clinical examinations

Systolic blood pressure (SBP, mmHg), diastolic blood pressure (DBP, mmHg), and heart rate [beats per minute (bpm)] were measured as systemic parameters. We also measured intraocular pressure (IOP, mmHg), axial length (AL, mm), cycloplegic SE, and BCVA as ocular parameters. IOP and cycloplegic SE were measured using a TONOREF 2™ device (Nidek, Aichi, Japan) and AL was measured using an optical biometer OA1000™ (Tomey, Aichi, Japan). The BCVA was measured at a 5 m distance. The mean arterial blood pressure (MABP, mmHg) and ocular perfusion pressure (OPP, mmHG) were calculated using the following formulas. MABP formula: DBP + (SBP – DBP)/3. OPP formula: (2/3MABP) – IOP.

### 2.5. Statistical analysis

All statistical analyses were performed using JMP ver. 14 software (SAS Institute, Cary, NC, USA). Chi-square tests were used to compare sex. The paired *t*-test was used to compare differences between the amblyopic and fellow eyes, and a non-paired *t*-test was used to compare differences between amblyopic and fellow eyes and control eyes. The correlation between the AL and SE and blood flow was analyzed by Pearson's correlation coefficient. All measurement values are expressed as the mean ± standard deviation (SD), and *p* < 0.05 were considered significant.

## 3. Results

In the control group, of the eight patients, three were measured only in one eye due to a lack of cooperation. Thus, the control group included 13 eyes of 16 possible eyes from the 8 pediatric control patients. Tables 1, 2 present the demographic data and clinical parameters. The SEs of amblyopic, fellow and control eyes were 4.91 ± 1.49, 1.81 ± 1.36, and 3.30 ± 2.03D, respectively. The difference in SE between amblyopic and fellow eyes was 3.11 ± 0.81D. The SEs in three groups differed significantly (amblyopic eye vs. fellow eye: *P* < 0.0001, paired *t*-test; control eye vs. amblyopic eye and fellow eye: *P* < 0.05 for both comparisons, non-paired *t*-test). The AL of the amblyopic, fellow, and control eyes was 21.41 ± 0.93, 22.46 ± 0.96 and 21.52 ± 0.60 mm. The AL in the fellow eye was significantly longer than that in the amblyopic and control eyes (*P* < 0.01 for both comparisons).

**Table 1:** Demographics and clinical characteristics of the study sample.

	**Anisometropic amblyopia group (*n* = 19)**	**Control group (*n* = 8)**	***P*-value**
Age, years	8.2 ± 3.0 (7.2–9.2)	6.8 ± 1.9 (5.7–8.0)	0.2040
Men: women	12:7	5:3	0.9742
SBP, mmHg	96.9 ± 10.2 (93.6–100.3)	91.4 ± 7.9 (86.6–96.1)	0.1460
DBP, mm Hg	61.1 ± 11.4 (57.4–64.9)	59.3 ± 11.7 (52.3–66.3)	0.9094
MABP, mmHg	73.1 ± 10.5 (68.3–77.7)	70.0 ± 9.0 (64.3–75.6)	0.4058
Heart rate, bpm	83.2 ± 15.9 (75.5–90.8)	87.0 ± 8.6 (79.8–94.2)	0.5286

The data are presented as mean ± standard deviation (95% confidence interval). SBP, systolic blood pressure; DBP, diastolic blood pressure; MABP, mean arterial blood pressure; bpm, beats per minute.

**Table 2:** Comparison of the SE, axial length, IOP, and OPP values among the amblyopic, fellow, and control eyes.

	**Amblyopic eyes (*n* = 19)**	**Fellow eyes (*n* = 19)**	**Control eyes (*n* = 13)**	***P*-value (amblyopia vs. fellow)**	***P*-value (amblyopia vs. control)**	***P*-value (fellow vs. control)**
Spherical equivalent (SE, D)	4.91 ± 1.49 (4.19–5.63)	1.81 ± 1.36 (1.15–2.46)	3.30 ± 2.03 (2.07–4.52)	< 0.0001	0.0144	0.0184
Axial length (mm)	21.41 ± 0.93 (20.95–21.85)	22.46 ± 0.96 (21.99–22.91)	21.52 ± 0.60 (21.16–21.88)	< 0.0001	0.6908	0.0040
IOP, mmHg	14.2 ± 2.3 (13.1–15.3)	14.1 ± 2.7 (12.8–15.4)	15.5 ± 1.6 (14.6–16.5)	0.7899	0.059	0.0999
OPP, mmHg	34.5 ± 7.3 (31.0–38.0)	34.6 ± 8.0 (30.8–38.4)	31.3 ± 4.9 (28.2–34.1)	0.7899	0.0791	0.1742

The data are presented as mean ± standard deviation (95% confidence interval). IOP, intraocular pressure; OPP, ocular perfusion pressure; spherical equivalent (SE).

### 3.1. Blood flow

Table 3 shows the MBR, ARBS, and sample size results. The MBR-As of amblyopic, fellow, and control eyes were 26.1 ± 3.6, 23.4 ± 3.5 and 22.6 ± 3.5, respectively. The MBR-A of the amblyopic eye was significantly higher than that of the fellow and control eyes (*P* = 0.0001 and *P* = 0.0108, respectively, for both comparisons). The MBR-Ts of the amblyopic, fellow, and control eyes were 11.4 ± 2.1, 10.3 ± 1.7, and 10.9 ± 1.1, respectively. The MBR-T of the amblyopic eye was significantly higher than that of the fellow eye (*P* < 0.05). The MBR-V did not differ significantly. The ARBS values of amblyopic, fellow, and control eyes were 40.9 ± 6.7, 36.7 ± 6.2, and 34.9 ± 5.9%, respectively. The ARBS of the amblyopic eye was significantly higher than that of the fellow and control eyes, indicating the ratio of vessel components in the amblyopic eyes was higher than that in the fellow and control eyes (*P* < 0.05). The MBR-choroid did not differ significantly among the three groups. MBR-A, MBR-T, and MBR-V were not significantly correlated with SE and AL.

**Table 3:** Comparison of the MBR, ARBS, and sample size among the amblyopic, fellow, and control eyes.

	**Amblyopic eyes**	**Fellow eyes**	**Control eyes**	***P*-value (amblyopia vs. fellow)**	***P*-value (amblyopia vs. control)**	***P*-value (fellow vs. control)**
MBR-A	26.1 ± 3.6 (22.4–27.8)	23.4 ± 3.5 (21.7–25.0)	22.6 ± 3.5 (20.5–24.8)	0.0001	0.0108	0.5554
MBR-V	47.4 ± 5.4 (44.8–45.0)	46.0 ± 6.1 (43.0–38.9)	43.9 ± 5.8 (40.1–47.5)	0.1287	0.0981	0.3620
MBR-T	11.4 ± 2.1 (10.4–12.4)	10.3 ± 1.7 (9.5–11.1)	10.9 ± 1.1 (10.3–11.6)	0.0154	0.4585	0.2273
ABRS (%)	40.9 ± 6.7 (37.7–40.1)	36.7 ± 6.2 (33.7–39.7)	34.9 ± 5.9 (31.3–38.5)	0.0139	0.0149	0.4333
Sample-A	37,413 ± 6,154 (34,447–40,379)	39,198 ± 7,873 (35,403–42,993)	42,858 ± 8,265 (37,863–47,852)	0.1344	0.0408	0.2153
Sample-V	15,202 ± 3,356 (13,584–16,819)	14,411 ± 3,664 (12,644–16,177)	15,700 ± 4,313 (13,093–18,036)	0.3326	0.7161	0.3702
Sample-T	22,211 ± 4,641 (19,975–24,448)	24,438 ± 5,773 (21,655–27,220)	26,769 ± 7,655 (22,143–31,395)	0.0378	0.0442	0.3335
MBR-choroid	4.0 ± 1.5 (3.3–4.7)	4.2 ± 1.1 (3.7–4.8)	4.0 ± 1.2 (3.3–4.7)	0.7501	0.8002	0.9126

The data are presented as mean ± standard deviation (95% confidence interval). MBR, mean blur rate; ARBS, area ratio of the bloodstream; MBR-choroid, choroidal mean blur rate; MBR-A, mean of all values in optic nerve head; MBR-V, vessel mean; MBR-T, tissue mean; Sample-A, mean of all sample sizes; Sample-V, vessel mean; Sample-T, tissue mean.

The sample numbers, which represent the size of the optic nerve, reflected in Sample-A of amblyopic, fellow, and control eyes were 37,413 ± 6,154, 39,198 ± 7,873, and 42,858 ± 8,265, respectively. Sample-A in the amblyopic eye was significantly higher than that in the control eye. The Sample-Ts of amblyopic, fellow, and control eyes were 22,211 ± 4,641, 24,438 ± 5,773, and 26,769 ± 7,655, respectively. Sample-T of the amblyopic eye was significantly smaller than those of the fellow and control eyes (*P* < 0.05, for both comparisons). Sample-V did not differ significantly among the amblyopic, fellow, and control eyes.

### 3.2. Reproducibility

Table 4 provides the COVs and ICCs for the MBR, sample, ARBS, and pulse waveform parameters in the ONH. The COVs for the MBR, sample, and ARBS were all ≤10%, and the ICCs were all ≥0.7. Among the pulse waveform parameters, the COVs of fluctuation, BOS, BOT, and RI were ≤10%. The ICCs of all pulse waveform parameters were <0.7, except for fluctuations.

**Table 4:** Coefficient of variation and intraclass correlation coefficient for blood flow values, number of sample, ARBS, and wave form parameters in the optical nerve head.

	**Average**	**COV**	**ICC**
Sample-A	39,466 ± 7,547 (37,343–41,589)	0.3 ± 0.9	1.00
Sample-V	15,034 ± 3,692 (13,996–16,072)	9.7 ± 8.4	0.71
Sample-T	24,203 ± 6,087 (22,491–25,915)	9.0 ± 18.0	0.83
MBR-A	24.2 ± 3.8 (23.2–25.3)	5.9 ± 3.5	0.82
MBR-V	46.0 ± 5.8 (44.3–47.6)	6.3 ± 3.9	0.71
MBR-T	10.9 ± 1.8 (10.4–11.4)	6.7 ± 5.6	0.79
ARBS	37.8 ± 6.7 (35.9–39.7)	8.4 ± 5.5	0.71
FLuctuation	11.1 ± 2.4 (10.4–11.8)	9.1 ± 5.6	0.71
Skew	8.0 ± 1.5 (7.6–8.5)	20.5 ± 18.5	0.23
BOS	80.6 ± 3.7 (79.5–81.6)	2.6 ± 1.7	0.67
BOT	62.6 ± 3.4 (61.7–63.6)	6.1 ± 3.8	0.16
Rising rate	13.4 ± 1.5 (12.9–138)	11.0 ± 8.7	0.27
Falling rate	11.0 ± 0.8 (10.8–11.2)	11.3 ± 11.3	0.07
FAI	3.5 ± 0.9 (3.2–2.7)	16.7 ± 11.9	0.52
ATI	31.6 ± 5.4 (30.1–33.1)	16.0 ± 11.5	0.32
RI	0.30 ± 0.32 (0.28–0.32)	9.8 ± 6.2	0.65

The data are presented as mean ± standard deviation (95% confidence interval). COV, coefficient of variation; ICC, intraclass correlation coefficient; MBR-A, mean of all values; MBR-V, vessel mean; MBR-T, tissue mean; Sample-A, mean of all sample sizes; Sample-V, vessel mean; Sample-T, tissue mean; ARBS, area ratio of the blood stream; BOS, blowout score; BOT, blowout time; FAI, flow acceleration index; ATI, acceleration time index; RI, resistivity index.

The results of COV and ICC by MBR-choroid showed the same trend as the reproducibility in the ONH. The COV for the MBR choroid were ≤10%, and the ICC were ≥0.7. Among the pulse waveform parameters, the COVs of BOS, BOT, falling rate, and RI were ≤10%. The ICCs of all pulse waveform parameters were <0.7, except for the FAI.

## 4. Discussion

The findings of the present study demonstrate that measuring of ocular blood flow in pediatric patients using LSFG was reproducible to the same degree as for adults. In the ONH and choroid, the COVs of all MBR values, sample size, ARBS, and even pulse waveform parameters such as the BOS, BOT, and RI were ≤10%. The ICCs of all MBRs, sample sizes, and ARBS scores were ≥0.7. In the ONH, the MBR-A of the amblyopic eye was significantly higher than that of fellow and control eyes. The MBR-T score of the amblyopic eye was significantly higher than that of the fellow eye. Sample-A of the amblyopic eye was significantly smaller than that of the control eye, and Sample-T was also significantly smaller than that of the fellow and control eyes. The ARBS was significantly higher in the amblyopic eyes than in the fellow and control eyes. Thus, the amblyopic eyes showed higher blood flow and smaller ONH size than the control eyes, but amblyopic and control eyes were not significantly different in AL.

This is the first study to confirm the reliability of ocular blood flow measurements using the LSFG in pediatric patients. In previous reproducibility studies in adult patients with glaucoma, the COVs ranged from 0.9 to 3.8% and the ICCs ranged from 0.95 to 0.98 (14). The COVs in patients measured in a supine position during surgery ranged from 3.1 to 6.9% (25); the COV for those in an upright position after being in a supine position was 6.7% (26), and the COVs in neonates ranged from 7.7 to 9.7% (23). In the present study, the reproducibility of the MBR in the ONH was 5.9%, which was very close to that observed in studies of adult patients; due to reproducibility with an ICC of ≥0.7, our results suggest sufficient reliability of LSFG for clinical use.

In this study, reproducibility in terms of both the COV and ICC was not favorable regarding the skew, rising rate, falling rate, FAI, or ATI. Large deviations in the COVs and ICCs were observed in the BOS, BOT, rising rate, and falling rate. According to Tsuda et al., pulse waves such as those in fluctuation, skew, and FAI in ocular blood flow are highly sensitive to subtle changes (27). In a study of neonates using LSGF-baby, Matsumoto et al. reported that reproducibility of COVs in pulse waves such as those in the fluctuation, skew, FAI, and RI could not be achieved; in pulse waves such as those in the BOS, BOT, rising rate, and falling rate, deviations in COVs and ICCs similar to those obtained in the present study were observed (23). The likely reason for this may be that children have higher heart rates than adults, making them prone to subtle changes in sight lines and body movements at the time of measurement.

The ONH in the amblyopic eye was significantly smaller than that in the fellow and control eyes. Because the ONH sample size of the vessels was not significantly different, the difference in size of the ONH was attributed to the difference in size of the tissue. In fact, the size of the tissue in the amblyopic eye was significantly smaller than that in the fellow and control eyes, and the ARBS representing the proportion of vessels in the amblyopic eye was significantly higher than that in the fellow and control eyes. Some researchers have reported that the size of the ONH in anisometropic eyes is significantly smaller than that in fellow or control eyes (28–30). The results of the present study are consistent with these findings. Lempert speculated that optic nerve hypoplasia leads to a decrease in ONH size in amblyopic eyes and associated retinal nerve fiber layer (RNFL) thinning, which impairs the anterior visual pathway and reduces visual function (5). However, Huynh and Wang reported that ONH size and RNFL thickness are associated in children, resulting in a small ONH that tends to thin the RNFL (31). The thickness of the RNFL varies depending on the refractive error and axis length, and some reports have shown that there is no significant difference between the thickness of the RNFL in the amblyopic eye and the fellow eye, while others have reported that the amblyopic eye has a thicker RNFL (32–34). There are no reports of RNFL thinning in amblyopic eyes, and the fact that the size of the ONH in anisometropic amblyopic eyes is smaller than in fellow eyes and normal eyes is a structural feature of anisometropic amblyopic eyes rather than optic nerve hypoplasia.

In the current study, the amblyopic eye had a significantly higher MBR-A than the fellow and control eyes in the ONH group. Some past studies that compared retrobulbar blood flow, that is, ophthalmic artery and central retinal artery, in amblyopic and fellow eyes reported that retrobulbar blood flow did not differ significantly between amblyopic and fellow eyes, indicating that the blood flow supplied to the anisometric amblyopic eye and the fellow eye with different axial lengths are the same (8, 9). Kobayashi et al. reported that, in normal eyes, blood flow in the ONH measured by LSFG did not differ significantly between eyes (35). Therefore, the reason for the higher MBR-A in the amblyopic eyes in the current study is that the size of the tissue component in the ONH of the amblyopic eyes was significantly smaller, while the size of the vascular component did not differ significantly among eyes. As a result, the same amount of blood flow passed through the smaller tissue component, resulting in higher blood flow velocity in the tissue component and faster overall blood flow velocity in the ONH. The vascular density of the ONH measured by optical coherence tomography angiography (OCTA) was significantly lower in amblyopic eyes than in fellow eyes, which is different from the present result that there was no significant difference in the size of the vascular component between amblyopic eyes and fellow or normal eyes (36). This discrepancy is because Sobral et al. enrolled patients with strabismic and anisometropic amblyopia, whereas we enrolled patients with anisometropic amblyopia without strabismus (36). Moreover, they included children who had been treated but had not reached 20/20, whereas we enrolled amblyopic eyes whose visual acuity had reached 20/20 or better with treatment. In addition, the difference in the analysis method between OCTA and LSFG may also have affected this discrepancy, as OCTA analyzes the superficial vascular structure, but LSFG analyzes blood flow from the superficial layer to the area around the stromal plate (37, 38). Although MBR measured by LSFG was significantly correlated with peripapillary relative intensity (PRI) and circumpapillary vessel density (spVD) by OCT A, OCTA has an advantage of detecting visualization of vascular structure in each layer, while LSFG (MBR and 9 pulse wave parameters) has an advantage of assessing physiological phenomenon such as vascular resistance and auto regulation of retinal microvascular circulation and defocus (39–43).

There was no significant difference in choroidal blood flow in this study. Hashimoto et al. reported by case report that, although the MBR was decreased in the amblyopic eye before treatment, the MBR increased with improvement in visual acuity after treatment, and the difference in MBR between both eyes became smaller (21). Some researchers have reported that the choroidal thickness of the amblyopic eye is greater than that of normal eyes with the same axial length or fellow eyes. In a study that focused on the structure of the choroid, that is, lumen and stroma, the lumen was larger, the stroma was smaller, and the ratio of lumen/stroma was larger in amblyopic eyes before treatment than in fellow and normal eyes; however, after treatment, the lumen and stroma became smaller and larger, respectively, and the ratio of lumen/stroma was the same as that in fellow and normal eyes (44). Changes in the choroidal structure that occur during treatment may affect choroidal blood flow. In this study, we analyzed choroidal blood flow in eyes with anisometropic amblyopia in which visual acuity was improved by treatment, resulting in the absence of significant differences among amblyopic, fellow, and normal eyes.

The present study had some limitations. First, no comparison between fundus photographs and the ONH area using OCTA could be performed. Instead, we calculated the area based on the sample size of the ONH. The sample size did not consider refraction and axial length, and further studies on sample size values are needed. Second, although choroidal blood flow may reflect choroidal structure, visual function, and pathophysiology of anisometropic amblyopia, in this study, choroidal blood flow was analyzed between the macula and ONH, but not in the macula, because multiple locations could not be measured due to insufficient cooperation for the examination. In the ONH, MBR was not significantly correlated with AL or SE. The ONH structure may have influenced this finding. Moreover, although past studies have investigated correlations between AL or SE and blood flow in patients with myopia and hyperopia, they have excluded amblyopia, whereas we enrolled patients with hyperopia and hyperopic anisometropic amblyopia. It was thought that these things influenced the result which was not indicate correlation. Thus, the relationship between ONH and choroidal structure, visual function, and blood flow in the ONH and macula should be investigated in the future by increasing the number of patients. Third, inter-examiner reproducibility could not be studied due to insufficient patient cooperation for the LSFG examination. In the future, inter-examiner reproducibility should be examined. Fourth, in this study, although we measured blood flow in pediatric patients, we need to investigate the blood flow not only pediatric patients but also adult patients with anisometropic amblyopia for improving the reliability of this study. Fifth, the number of subjects in this study was small. Because there was no report of evaluating in blood flow using LSFG in the anisometropic amblyopic eye excluding case report, we calculated sample size using previous report investigating ONH size among amblyopic, fellow and control eye. The size of ONH in each group was 2.57, 1.74, 1.55 mm, respectively. We calculated the sample size based on this previous study, and at least 28 eyes were required in total for this study design (α = 0.05, power 80%), indicating that the 49 eyes enrolled in this study constitute a reasonable sample size, but the sample size seems small when considered as a study of blood flow. Because we conducted this study retrospectively, in further study we prospectively need to investigate blood flow in enough number of patients with anisometropic eye (29).

## 5. Conclusion

In conclusion, we were able to measure ocular blood flow in pediatric patients, and our results suggest that good reproducibility was achieved for clinical use. Moreover, the MBR values of the ONH in amblyopic eyes were high, and ocular structural differences were observed. It has been suggested that measuring ONH blood flow using LSFG could detect ocular structural changes in anisometropic amblyopic eyes.

## Data availability statement

The original contributions presented in the study are included in the article/supplementary material, further inquiries can be directed to the corresponding author.

## Ethics statement

The studies involving human participants were reviewed and approved by Institutional Review Board of Toho University Omori Medical Center (#M22161). Written informed consent from the participants' legal guardian/next of kin was not required to participate in this study in accordance with the national legislation and the institutional requirements.

## Author contributions

TI and TM: design of the study. TI and MK: collection of data, management, analysis, and interpretation of the data. TI, TM, SM, and YH: preparation and review of the manuscript. All authors contributed to the article and approved the submitted version.
